# A systematic approach re-analyzing the effects of temperature disturbance on the microbial community of mesophilic anaerobic digestion

**DOI:** 10.1038/s41598-019-42987-0

**Published:** 2019-04-25

**Authors:** Grace Tzun-Wen Shaw, Chieh-Yin Weng, Cheng-Yu Chen, Francis Cheng-Hsuan Weng, Daryi Wang

**Affiliations:** Biodiversity Research Center, Academia Sinica, Taipei, 115 Taiwan

**Keywords:** Environmental biotechnology, Network topology, Metagenomics

## Abstract

Microbial communities are key drivers of ecosystem processes, but their behavior in disturbed environments is difficult to measure. How microbial community composition and function respond disturbances is a common challenge in biomedical, environmental, agricultural, and bioenergy research. A novel way to solve this problem is to use a systems-level perspective and describe microbial communities as networks. Based on a mesophilic anaerobic digestion system of swine manure as a tool, we propose a simple framework to investigate changes in microbial communities via compositions, metabolic pathways, genomic properties and interspecies relationships in response to a long-term temperature disturbance. After temperature disturbance, microbial communities tend towards a competitive interaction network with higher GC content and larger genome size. Based on microbial interaction networks, communities responded to the disturbance by showing a transition from acetotrophic (*Methanotrichaceae* and *Methanosarcinaceae*) to methylotrophic methanogens (*Methanomassiliicoccaceae* and *Methanobacteriaceae*) and a fluctuation in rare biosphere taxa. To conclude, this study may be important for exploring the dynamic relationships between disturbance and microbial communities as a whole, as well as for providing researchers with a better understanding of how changes in microbial communities relate to ecological processes.

## Introduction

Environmental disturbances are important factors in shaping the structure of plant, animal or microbial populations. In the past few decades, remarkable progress has been made towards understanding how disturbance affects microbial composition, biodiversity loss and ecological processes^1–5^. Responses by microbial communities to disturbed environments over time are critical to consider in the context of driving key ecosystem processes. In general, resistance and resilience are two important drivers that stabilize microbial communities after a disturbance^6,7^. In recent years, there has been a dramatic proliferation of research concerned with the issue of whether microbial communities stay resistant to disturbance, where various disturbances from experimental designs or environmental events were considered. The majority of these studies had a long-term experimental period and had concluded that the microbial composition was sensitive to disturbance, such as CO_2_ enrichment^1^, fertilization with mineral nutrients^2^, enrichment with C substrates^3^, or climate change^4,5^. Together, these studies have provided insights into the temporal scale at which community turnover and disturbance responses contributed to similar ecological systems. Further investigation is needed to distinguish between the functional redundancy and specificity of microbial communities to help explore the microbial roles under a similar or dissimilar ecological processes.

Microbial functions in various environments have recently drawn great attention due to the introduction of next generation sequencing (NGS) technologies^6^. Microbial communities harboring a high degree of compositional or functional changes may correspond with changes in population niche or genomic properties, including guanine-cytosine (GC) content or genome size^8,9^. Numerous experiments have indicated that an increased GC content is associated with an aerobic lifestyle^10^, large genome size^11,12^, variable environments^11^, or disturbance^4^. Furthermore, many researches in recent years have focused on recognizing the roles of competition and disturbance in shaping ecological communities: interspecies competition may stabilize the entire ecosystem^13^, but is sensitive to disturbance^14^. However, one common pattern in these researches is that they considered only a single biological feature, for example, effects of a disturbance on changes in microbial community^15^, genomic properties^16^ or microbial interactions^14^, which may be intertwined with the response to environmental changes. These independent studies make it difficult to systematically decipher the influence of disturbance on the different identities of a whole microbiome simultaneously. The aim of this paper, therefore, is to investigate the effect of a press disturbance on microbial composition, function, genomic properties, and microbial interactions as a whole. This analyzing pipeline may provide a new insight or alternative solution to the problem of an overall and deep understanding of microbial composition change, functional shift, genomic features, specific interactions and topological niche under a pulse or press disturbance.

Ideally, an anaerobic digestion (AnD) system may serve as a model experiment for investigating a pulse or press disturbance for a short-term duration^15,17^. In previous studies, resilient or altered microbial communities were observed after a two-day or one-week temperature shock^15,17,18^. In short, in this study the AnD of swine manure was performed at the mesophilic temperature (37 °C) for two months with an eight-day temperature shock to mimic the condition of a long-term press disturbance. To systematically examine the effects of disturbance on the whole microbiome, such as composition, metabolic function, genomic properties or interspecies competition of microbial communities, we will apply the recent innovations in microbial technologies, such as 16S-rRNA sequencing, bioinformatics, and network inference, to overcome the major obstacles using traditional approaches. In this study, we used a mesophilic AnD system of swine manure to investigate the effects of a long-term temperature disturbance on dynamic microbial communities from a systems-level perspective. After the temperature disturbance, microbial communities shifted to a competitive interaction structure with higher GC content and larger genome size. We observed the topological transition of acetotrophic (*Methanotrichaceae* and *Methanosarcinaceae*) to methylotrophic methanogens (*Methanomassiliicoccaceae* and *Methanobacteriaceae*) and the fluctuation of rare biosphere taxa against disturbance. Results of this study could have a considerable impact on realizing the roles that microbial communities play under a long-term stress in ecology and will serve as the foundation for system control to maintain stable AnD process.

## Materials and Methods

### Inoculum and substrate

The inoculum, also called seeding sludge, used in this study was obtained from a mesophilic full-scale biogas plant located in Miaoli County, Taiwan. Swine manure, the primary substrate of anaerobic digesters, was collected from DaXi pig farm in Miaoli County, Taiwan; we received approval from the farm owner to collect the concentrated pig manure. After collection, the inoculum was stored in a room temperature condition, and swine manure was kept frozen in a −20 °C freezer and thawed before use.

### Anaerobic digester operation

An anaerobic bench-top CSTR (completely stirred tank reactors) reactor (FS-02 series, Winpact, USA) with a working volume of 2.5 L was set up for this study^19^. The digester was operated at mesophilic (37 °C) conditions using 10 days hydraulic retention time (HRT), 5% total solids (TS), and a stirring speed of 90 rpm without pH control. At the beginning, the reactor was filled with the inoculum (1 L) and swine manure (1.5 L). After three days of digestion, 167 ml of influents (swine manure with 5% TS load) was daily fed into the AnD reactor with the addition of nitrogen gas and with a semi-continuous feeding mode. Once steady state was achieved in the reactor, its temperature in the reactor was changed to 23–25 °C from day 25 to 32 and swine manure was fed into it daily. After eight days of operation under lower temperature condition, the temperature of the AnD reactor was recovered to 37 °C at day 33 and swine manure was daily fed into the reactor until new steady state was achieved. During the operation of the CSTR reactor, all effluents were collected every day and stored in a −20 °C freezer for the subsequent analysis, such as DNA extraction and polymerase chain reaction (PCR) amplification of the 16S rRNA gene with primers 341F and 806R^20^.

To purge air from the AnD digester, a peristaltic pump (Masterflex model No. 7553-80, Cole-Parmer Instrument Co., IL, USA) was used to feed influents and pump effluents. TS and chemical oxygen demand (COD, colorimetric method) of influents and effluents were analyzed according to the American Public Health Association^21^. The level of pH, ORP (oxidation-reduction potential) and temperature, the production of biogas (GPR) or methane (MPR), and methane content (CH_4_%) were automatically detected by the CSTR fermentation system mentioned above (FS-02 series, Winpact, USA).

### Sampling strategy

Effluent samples were collected from the CSTR anaerobic reactor for microbial analysis before, during and after the temperature disturbance. From start-up of an AnD reactor to the operation of temperature disturbance (day 25), 12 effluent samples were collected at day 2, 6, 8, 10, and 13–20, denoted by T1-T12 for day 2 to 20. During the period of lowering temperature operation, three samples were collected at day 27, 29 and 31, denoted by T13-T15. After temperature of the AnD reactor was recovered to 37 °C, effluent sampling was manipulated at day 40, 42, 45, 50, 53, and 58–67 for T16-30.

### DNA extraction

All genomic DNA from the collected samples was extracted using PowerSoil^®^ DNA Isolation Kit (Mo Bio Laboratories, USA) according to the manufacturer’s instructions. DNA concentrations in the extracts were determined using a Qubit fluorometer (Invitrogen, Life Technologies, Carlsbad, CA, USA) with Quant-iT dsDNA HS assay kit. Extracted DNA was quantified using a Nanodrop-1000 Spectrophotometer (Thermo Scientific, Wilmington, DE, USA). To prevent artificial contamination, all above-mentioned procedures were manipulated in a laminar flow cabinet.

### PCR amplification and sequencing of 16S rRNA

The extracted DNA was amplified with a modified 341F (CCTAYGGGRBGCASCAG) and a modified 806R (GGACTACNNGGGTATCTAAT)^20^ and fused with Illumina overhanging adapters. The two universal primers can amplify a DNA fragment of about 533 bp length flanking the V3 and V4 regions of the 16S rRNA gene^22^. PCR conditions were set based on a previous study^23^. To remove excess primer dimers and dNTPs, confirm and purify all PCR products, 2% agarose gel electrophoresis with Tris- acetate-EDTA (TAE) buffer (TOOLS, Taiwan), NucleoSpin^®^ Gel and PCR Clean-up (Macherey-Nagel GmbH & Co. KG, Düren, Germany) were used. The cleaned PCR products were quantified by the Nanodrop-1000 Spectrophotometer (Thermo Scientific, Wilmington, DE, USA). The purified amplicons were then sent out for barcoded library preparation, according to the Illumina standard protocol of 16S rRNA sequencing library preparation, and sequenced on a MiSeq platform with the reagent kit v3.

### Sequence processing

The quality of all paired-end sequences was monitored by FLASH software^24^, MOTHUR v.1.36.1^25,26^ and the UPARSE OTU (operational taxonomic units) analysis pipeline^27^. In brief, the merging sequences with inexact matches to the forward and reverse primers, lengths shorter than 375 bp, and homopolymers longer than eight nucleotides were defined as low-quality and removed in the subsequent analysis. Chimeras were removed from the data using UPARSE^27^. The taxonomic assignment of 16S rRNA sequencing clusters, i.e. OTUs, from the superkindom level to genus level was based on a Ribosomal Database Project (RDP) classifier with a confidence score threshold of 80%, which was conducted by the classify.seqs script using the trainset14_032015.rdp in MOTHUR^25^. Finally, OTUs assigned to Chloroplast, Mitochondria, Eukaryota, or unknown kingdom were removed to generate an OTU table, and then taxonomical abundances were corrected by 16S rRNA gene copy number adjustment^28^. The detailed parameter settings are available in a previous study^23^. All sequenced samples were deposited in the NCBI Short Read Archive under BioProject PRJNA484128 (SRR7640822- SRR7640851).

### Classification of microbial members

An abundance profile of microbial communities can be assigned at different taxonomic levels. In this study, an abundance table at the family level was used for subsequent analysis. Each microbial family could be classified three times according to different classifying criteria, such as the level of abundance, the present densities, and abundance pattern, all described below.

The first classification of a microbial member was based on the level of abundance. A high-abundance (HA) microbial member contained on average more than 1% of the total number of sequences across time-series samples. If the averaged abundance of a microbial member among samples ranged from 0.1% to 1%, they were classified into the low-abundance (LA) group, and the remaining RA (rare in abundance) group. Next, based on present densities, a Core microbial member had abundance values, also defined as present, in all time-series samples, whereas a Ncore microbial member was defined to be present in partial samples, defined as three before or after temperature disturbance in this study. Finally, based on abundance patterns of a microbial member among samples, microbial members with a more stable or variable abundance pattern were defined as being stable (BS) or variable (BV), and the remaining as no change (NC) members. The primary criterion for determining abundance pattern of a microbial member is as follows.

For a microbial abundance table, there were *i* = 1, …, *N* microbial members and *k* = 1, …, *T* time-series samples where relative abundance was denoted by *x*_*ik*_. If there were *T*_1_ or *T*_2_ time-series samples before and after temperature disturbance, the mean value, coefficient of variation (CV) and an abundance pattern were defined by equations (1), (2) and (3), respectively, where variance test was conducted by F test of the equality of two variances with significant level of 0.05.

1 $${\bar{x}}_{i}^{k=1,\ldots ,T}=\frac{{\sum }_{k=1}^{T}({x}_{ik})}{number({x}_{ik} > 0)}$$

2 $$c{v}_{i}^{k=1,\ldots ,T}=\frac{\sqrt{\frac{1}{T-1}{\sum }_{k=1}^{T}{({x}_{ik}-{\bar{x}}_{i}^{k=1,\ldots ,T})}^{2}}}{{\bar{x}}_{i}^{k=1,\ldots ,T}}$$

3 $$\{\begin{array}{cc}BS: & if\,var\,({x}_{1},\ldots ,{x}_{T1})\ne \,var\,({x}_{T-T2+1},\ldots ,{x}_{T})\\  & and\,c{v}_{i}^{k=1,\ldots ,T1}\ge \,c{v}_{i}^{k=1,\ldots ,T2}\\ BV: & if\,var\,({x}_{1},\ldots ,{x}_{T1})\ne \,var\,({x}_{T-T2+1},\ldots ,{x}_{T})\\  & and\,c{v}_{i}^{k=1,\ldots ,T1} < \,c{v}_{i}^{k=1,\ldots ,T2}\\ NC: & if\,var\,({x}_{1},\ldots ,{x}_{T1})=var\,({x}_{T-T2+1},\ldots ,{x}_{T})\end{array}$$

### Induced or repressed microbial members during temperature disturbance

During the temperature disturbance, there were *T-T*_1_*-T*_2_ time-series samples in this study. If the abundance profile of a microbial member conveyed a convex (or concave) pattern among samples, the microbial member was called induced (or repressed) during temperature disturbance (equation (4)).

4 $$\{\begin{array}{cc}\begin{array}{c}induced:\end{array} & \begin{array}{c}{\bar{x}}_{i}^{k=T1+1,\ldots ,T-T2} > {\bar{x}}_{i}^{k=T1,\ldots ,T1}\,\\ {\bar{x}}_{i}^{k=T1+1,\ldots ,T-T2} > {\bar{x}}_{i}^{k=T-T2+1,\ldots ,T}\end{array}\\ \begin{array}{c}repressed:\end{array} & \begin{array}{c}{\bar{x}}_{i}^{k=T1+1,\ldots ,T-T2} < {\bar{x}}_{i}^{k=T1,\ldots ,T1}\,\\ {\bar{x}}_{i}^{k=T1+1,\ldots ,T-T2} < {\bar{x}}_{i}^{k=T-T2+1,\ldots ,T}\end{array}\end{array}$$

Furthermore, Shannon indices were constructed using the command *collect*.*single* in MOTHUR^25^ to compare the microbial diversity among time-series samples. Principal component analysis (PCA) was conducted by Matlab R2016a using the command *pca*. Before running PCA, microbial abundance table was transposed and standardized by the command *zscore*. All measurements in this study were expressed by a mean value with its standard error of the mean. Subsequently, two hypothetical tests were conducted below. At first, an F test for the equality of two variances was conducted to distinguish between samples before and after temperature disturbance with equal (P > 0.05) or uneqaul variance (P ≤ 0.05). Then, two sample Student’s one-tailed t tests with equal or uneqaul variance were used to calculate the significance before and after temperature disturbance.

### Assignment of metabolic pathways and genomic properties

For each microbial strain, metabolic pathway, genome size, GC content, and the number of genes or proteins in a genome were collected from KEGG (Kyoto Encyclopedia of Genes and Genomes)^29^ and NCBI (The National Center for Biotechnology Information) databases. Then, all functional or genomic information on each microbial species or strain was organized and averaged to specific taxonomic level (e.g. order, family or genus) based on our abundance table. Metagenome functional annotation for microbial abundance profiles was predicted by the PICRUST (Phylogenetic Investigation of Communities by Reconstruction of Unobserved States)^30^.

### Inferences from the microbial interaction network

For an abundance profile with *N* microbial members and *T* time-series samples, we randomly selected 90% of microbial members to infer microbial interactions using MetaMIS (Metagenomic Microbial Interaction Simulators)^31^ for 1,000 simulation times. There are two kinds of outcomes for a microbial interaction: positive or negative. A positive (or negative) interaction between two microbial members represents one microbe is capable of increasing (or reducing) the abundance of the other one. An interaction pair was considered to be reliable when tested by proportional tests to measure whether the proportion of negative (or positive) interactions is greater than 0.9. All reliable interaction pairs were collected and 10% of interaction pairs with strongest interaction strengths was selected for subsequent topological analysis.

### Topological inference

In a microbial network, a node indicates a microbial member and an edge represents an interactive relationship between two microbial members. We used Gephi software^32^ to calculate the topological niches of an interaction network. Two topological measurements, including in-degree centrality and betweenness centrality, were conducted. In-degree centrality counts how many neighbors a microbial member receives and is shown by node color, of which the darker (or lighter) red indicates a microbial member with more (or less) in-degree relationships. A larger value indicates that the microbial member is influenced by more neighbors or interactions. The betweenness centrality for each microbial member is the number of times that one member functions as a bridge along the shortest path between two other microbial members and displayed by node size. Therefore, a microbial member with a strong betweenness centrality has a bigger node size.

## Results

### Temperature disturbance drives different ecological processes

According to previous research findings^15^, eight-day temperature disturbance can be considered a long-term press disturbance to microbial communities. To realize how microbial communities are influenced by long-term temperature disturbance, six abiotic factors—removal rate of COD and TS, GPR, MPR, CH_4_% and ORP—and three biotic factors—microbial compositions, functions, and interactions—were measured and are discussed below.

#### The abiotic factors

**Figure 1:**
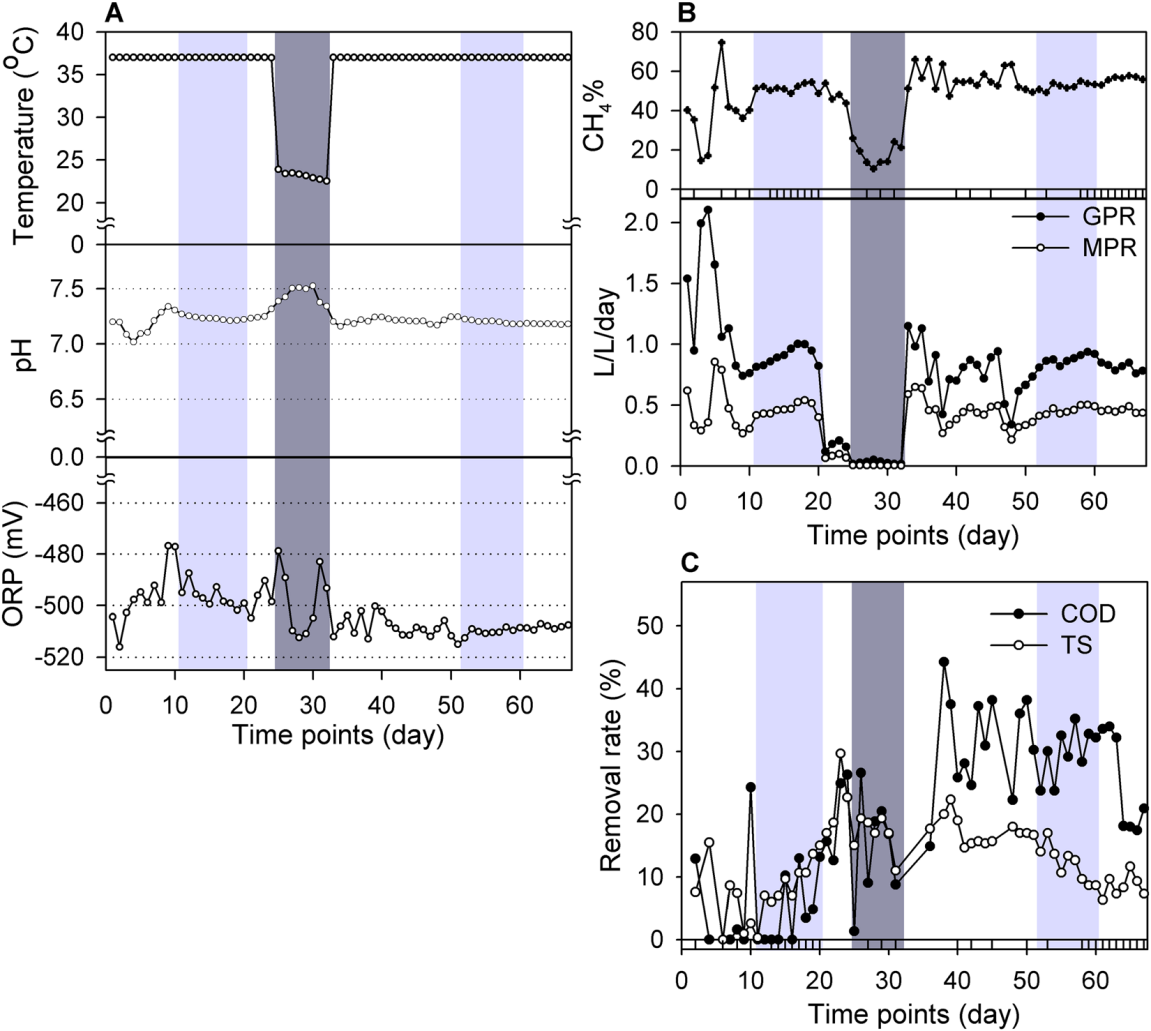
Reactor performances for the entire operational period. The change in (**A**) temperature, pH, ORP, (**B**) CH_4_%, GPR, MPR, and (**C**) removal rate of COD or TS are shown here. The dark grey region is the period of temperature disturbance from 37 °C to 23 °C, and the light grey region indicates reaching a steady state for biogas and methane production before and after temperature disturbance.

To mimic a long-term press disturbance, we operated a mesophilic (37 °C) anaerobic digester and then dropped to 23 °C for eight days after around 24 days of start-up operation (Fig. 1A). The monitoring profiles for temperature, pH, ORP, CH_4_%, GPR, MPR, and removal rate of COD and TS are shown in Fig. 1. The initial higher GPR (2.1 L/L/day at day 4) or MPR (0.85 L/L/day at day 5) (Fig. 1B) was due to the biogas and methane production from the undigested organics in the inoculum. There were two steady-state periods for the stable methane yields after around 10 or 51 days of operation (light grey regions in Fig. 1). Before the temperature decreased, the COD and TS removal efficiency steadily rose to 26.3% and 22.7% by day 24, respectively, as seen in Fig. 1C.

The operation of lowering the temperature from 37 °C to 23 °C made most abiotic factors—such as ORP (Fig. 1A), GPR, MPR, CH_4_% (Fig. 1B) and the removal rate of COD and TS (Fig. 1C)—decrease. Initially, the anaerobic rector had a stable daily production of biogas (0.94 ± 0.01) and methane (0.40 ± 0.04) before the temperature disturbance (Supplementary Table S1). After the sudden temperature decrease to 23 °C, the production of biogas and methane dropped to almost zero and resulted in a decrease in the methane content (CH_4_%) from 51.44% ± 0.68% (1^st^ steady state in the light grey region) to 17.84% ± 7.84%(dark grey region) (Fig. 1B). Lowering the temperature also reduced the removal efficiency of COD and TS from 26.3% and 22.7%, respectively, on day 24 to 8.78% and 11% at day 31 and was accompanied by a slight rise in ORP value from −505 mV on day 21 to −479 mV on day 25; ORP is a valuable parameter to monitor and control anaerobic fermentation status. Note that owing to a technical problem (stirring rate changed from 90 to 333 rpm at day 21), there was a decline in GPR and MPR from day 21. To clarify the effect, we repeated the AnD experiment (Supplementary Fig. S1), the results revealed a similar tendency with the observations in Luo *et al*.^15^. Therefore, we reasoned that the 16S rRNA data extracted from day 11 to day 20 (1^st^ steady state), and day 52 to day 60 (2^nd^ steady state) have reached a steady state (Fig. 1A) and are feasible for the following analysis.

As the temperature was raised from 23 °C to 37 °C at the 33th day, we observed a rapid recovery in pH value (7.2) (Fig. 1A) and a slow but sure return in GPR and MPR to achieve the second steady state after nineteen days of anaerobic fermentation from day 33 to 51 (Fig. 1B). At the same time, we observed the improved performance of CH_4_%, removal rate of COD and TS, and ORP. The CH_4_% from AnD digester was initially an average of 45.73% ± 2.48% before the temperature disturbance and then quickly increased to 55.06% ± 0.78% after the temperature increase from 23 °C to 37 °C (P = 6.34 × 10^−4^; Supplementary Table S1). After the second steady state of biogas and methane production was achieved, CH_4_% was progressively raised from 49.14% at day 53 to 57.68% at day 65, which conveyed a stable production of methane while the GPR was declining. Furthermore, the operation of temperature disturbance promoted the efficient removal rate of COD (or TS) from 7.76% ± 2.01% to 29.31% ± 1.36% (or from 10.36% ± 1.64% to 13.54% ± 0.79%) for the more consumption of swine manure, and displayed a more anaerobic environment for ORP levels from −496.47 ± 1.67 mV to −508.83 ± 0.53 mV (P = 6.18 × 10^−8^; Supplementary Table S1). All of the above abiotic factors may have contributed to the fact that temperature disturbance stimulated a more efficient utilization of organic matters and a more anaerobic condition for anaerobic digesters and implied a certain variation in microbial communities after temperature disturbance which was consistently observed in another replicate experiment (Supplementary Fig. S1).

#### The biotic factors

**Figure 2:**
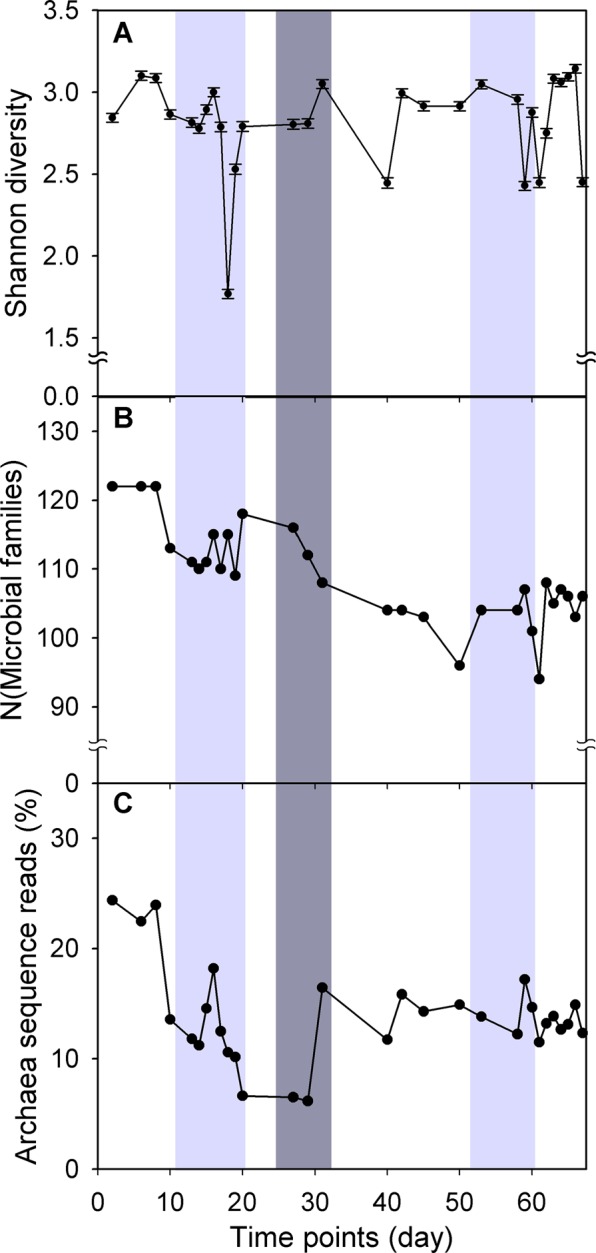
Robust microbial diversity and archaeal percentage. (**A**) Shannon diversity, (**B**) number of microbial families, and **(C)** archaeal percentage are listed.

**Figure 3:**
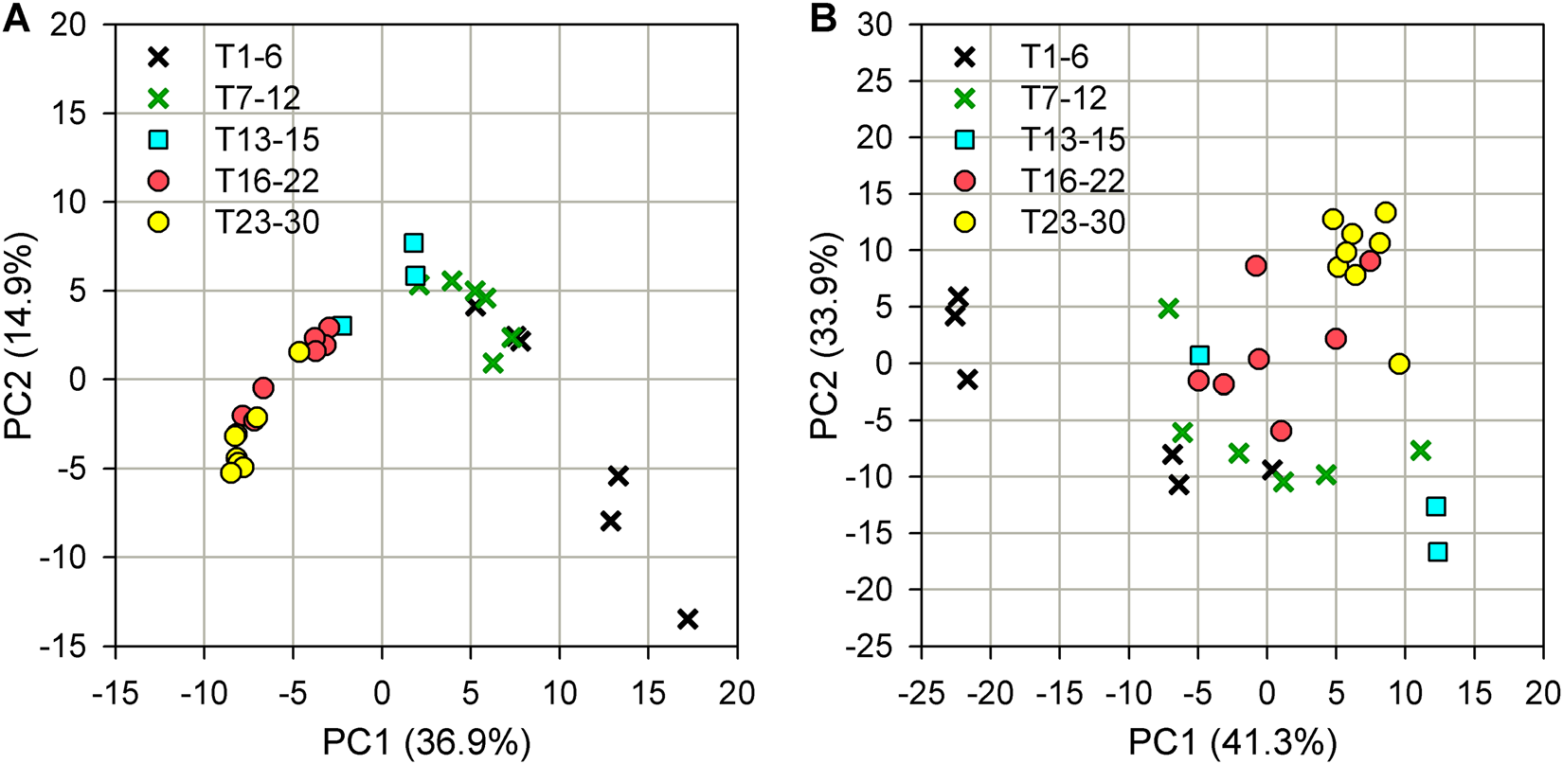
Different microbial composition and functional profile before and after temperature disturbance. PCA plot for (**A**) microbial abundance profiles and (**B**) functional profiles predicted by PICRUST^30^.

**Figure 4:**
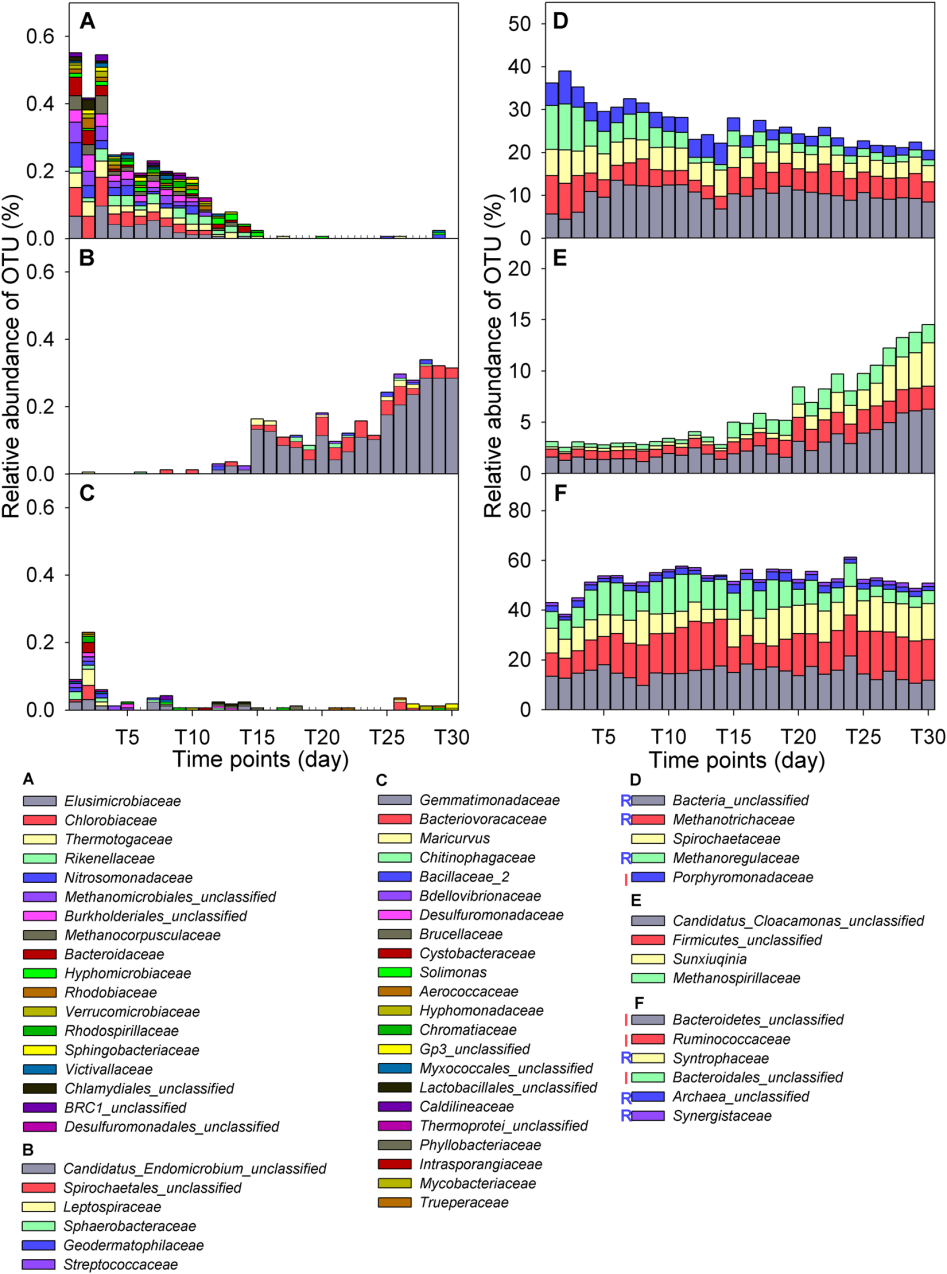
The dynamic microbial communities before and after temperature disturbance. Microbial composition changed dynamically and partitioned based on the following criteria: (**A**) Ncore/RA/BS, (**B**) Ncore/RA/BV, (**C**) Ncore/RA/NC, (**D**) Core/HA/BS, (**E**) Core/HA/BV, and (**F**) Core/HA/NC. All microbial members are at the family level.

A comprehensive understanding of the dynamic microbial communities was conducted by Shannon diversity index (Fig. 2 and Supplementary Table S1) and PCA (Fig. 3). The Shannon diversity index has been a popular diversity index in ecology and can account for both richness and evenness of microbial members simultaneously. A PCA plot was used to separate samples by different time intervals. Subsequently, all microbial families were exhaustedly assigned into twelve categories (Supplementary Table S2) according to their status as Core or Ncore member, level of microbial abundance (HA/LA/RA), and dynamic abundance pattern (BS/BV/NC) (Fig. 4, Supplementary Figs S2 and S3).

#### Change in microbial composition and function

Before the temperature decreased to 23 °C on day 25, the Shannon diversity index was 2.77 ± 0.10, which was similar with that after the temperature returned to 37 °C on day 33 (P = 0.28; Supplementary Table S1). Although biodiversity was similar after temperature disturbance (Fig. 2A), the number of microbial families decreased from 114.83 ± 1.45 to 103.47 ± 1.01 (Fig. 2B and Supplementary Table S1) and the proportion of archaeal populations was maintained at a constant level of 13.75% ± 0.41% (Fig. 2C and Supplementary Table S1). Apparently, a long-term temperature disturbance triggered the change in microbial composition. As expected, results (Fig. 3) from the PCA analysis were in good agreement with findings in Fig. 2: microbial composition during the early phase (T1-T5) was different with that during the final stage (T23-T30). Not only microbial composition (Fig. 3A) but also microbial function (Fig. 3B) was moved to a new status after temperature disturbance to drive different ecological processes. The details about the influence of temperature disturbance on each microbial member are illustrated in the next section.

#### The abundance pattern of each microbial member

We conducted three types of assignment for each microbial member based on the level of abundance (HA/LA/RA), Core or Ncore, and stability index (BS/BV/NC), 12 groups in total (Supplementary Table S2). There were totally 154 microbial families, including 46 members with partial presence (Ncore) and 108 with total presence (Core) in all time-series samples. The Ncore group only contained microbial families with RA pattern, but members in Core group can be further classified to nine different classes (Supplementary Table S2).

A great loss and a little gain in microbial members: At first, 46 Ncore/RA microbial families could be classified into three groups according to their abundance stability, i.e. BS, BV and NC (Fig. 4A–C). After temperature disturbance, 18 microbial families were dismissed (Fig. 4A), 6 microbial families were more abundant (Fig. 4B), and 22 microbial families (Fig. 4C) had similar abundance compared to abundance profiles before temperature disturbance. This observation was also shown in Core microbial families (Fig. 4, Supplementary Figs S2 and S3). In general, 43.5% of Core microbial families had invariant or unchanged abundance profiles among time-series samples (Fig. 4F, Supplementary Figs S2C and S3C). For the remaining (BS and BV; 56.5%), over half of them (BS; 62.3%) had less abundance after temperature disturbance (Fig. 4D, Supplementary Figs S2A and S3A). Only a small portion of the entire community increased its abundance level after the temperature came back to its original level (37 °C) (Fig. 4E, Supplementary Figs S2B and S3B).

Induced and repressed microbial members during temperature disturbance: Unlike Ncore microbial members, Core microbial members could be divided into three groups according to their differences in microbial abundances across three time intervals, T1-T12, T13-T15, and T16-30. The induced (or reduced) group, denoted by I (or R) (Fig. 4, Supplementary Figs S2 and S3), conveyed higher (or lower) abundance during the period of lower temperature compared with its abundance before or after temperature disturbance. In general, 53.7% of Core microbial members had induced (25.9%) or repressed (27.8%) abundances at 23 °C. The induced group was composed of four microbial families: *Porphyromonadaceae* for hydrolysis (Fig. 4D), *Bacteroidetes_unclassified*, *Ruminococcaceae*, and *Bacteroidales_unclassified* (Fig. 4F); the reduced group contained *Methanoregulaceae* and *Methanotrichaceae* for methanogenesis, *Bacteria_unclassified* (Fig. 4D), *Syntrophaceae* for acetogenesis, *Synergistaceae*, and *Archaea_unclassified* (Fig. 4F). For microbial members with rare or low abundances (Supplementary Figs S2 and S3), almost half of Core microbial members had induced or repressed pattern in the BV, BS, and NC groups, especially for a high proportion (66.7%) in Core/LA/BS group (Supplementary Fig. S3A). In a resilient microbial community, we can expect to observe that microbial abundance is suppressed (or induced) by a disturbance, but is able to recover to its original level quickly. However, in this study we observed that the microbial members with a disturbance-induced (or -repressed) abundance did not always decrease (or increase) in abundance after temperature disturbance. For example, *Fibrobacteraceae* and *Gracilibacteraceae* were two microbial families and had induced abundance in the temperature disturbed interval (T13-15). However, after temperature disturbance, the abundance of *Fibrobacteraceae* decreased but *Gracilibacteraceae* increased. Thus, under a long-term press disturbance, microbial communities were sensitive to disturbance and did not rapidly recover to their original statuses.

A shift to hydrogenotrophic methanogens after temperature disturbance: In considering the anaerobic-digestion issue, the first question that conventionally arises concerns what kind of methanogenic archaea enhances biogas or methane production. To that end, the abundance fluctuation of eight methanogens in this study was scrutinized under the AnD system. We observed a shift in hydrogenotrophic methanogens from *Methanocorpusculaceae* (Fig. 4A) and *Methanoregulaceae* (Fig. 4D) to *Methanomicrobiaceae* (Supplementary Fig. S2A) and *Methanospirillaceae* (Fig. 4E) after temperature disturbance, respectively. Furthermore, an acetotrophic methanogen (*Methanotrichaceae*, Fig. 4D), a methylotrophic methanogen (*Methanomassiliicoccaceae*, Supplementary Fig. S3A), and two methanogens with multiple carbon sources (*Methanosarcinaceae*, Supplementary Fig. S2A and *Methanobacteriaceae*, Supplementary Fig. S3C) revealed invariant or stable abundance profiles after temperature disturbance.

### Metabolic pathways, genomic properties and temperature disturbance

**Figure 5:**
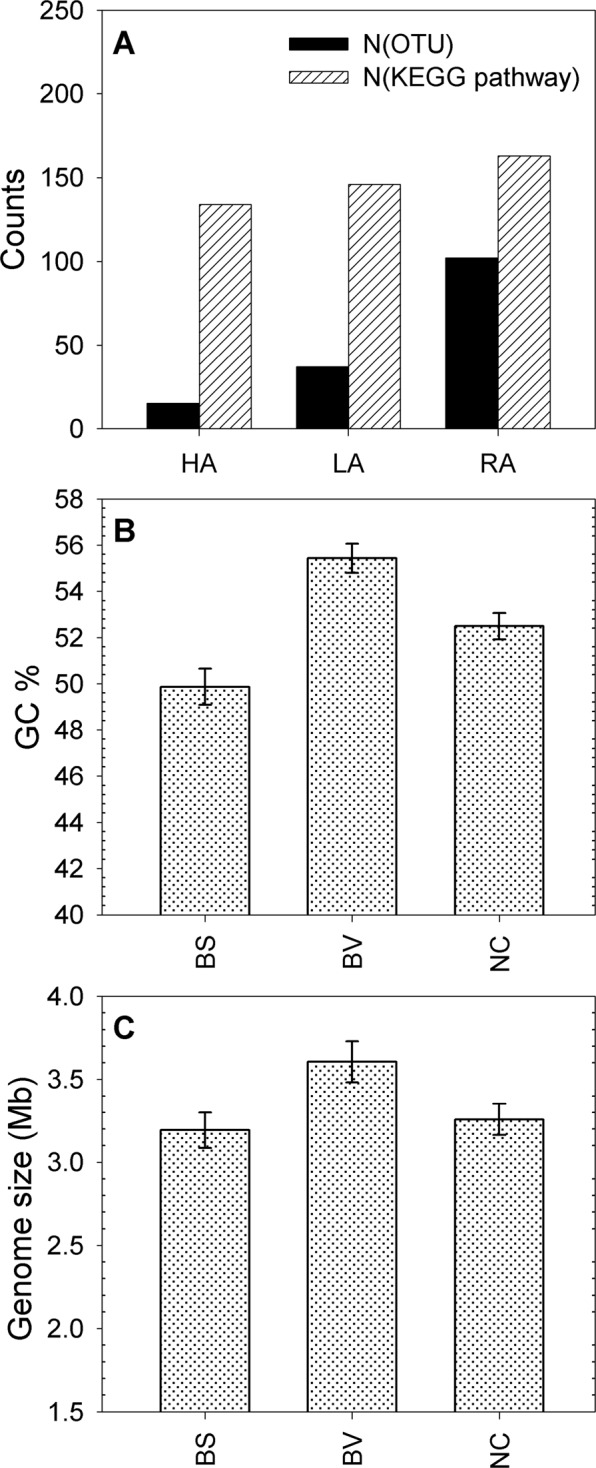
Metabolic pathaway and genomic properties at family level. (**A**) Number of microbial families or KEGG pathways for each abundance pattern (i.e. HA, LA and RA). (**B**) The GC content (GC%) and (**C**) genome size for groups with different abundance pattern (i.e. BS, BV and NC).

In order to understand what functional scenario is motivated by temperature disturbance, it is necessary to examine the change in metabolic function and genomic features of microbial communities from public domain databases^33,34^. In this study, 154 microbial families could be separated by their level of abundance—15 HA, 37 LA and 102 RA families—or by their abundance pattern—44 BS, 41 BV and 69 NC families and annotated with functional information, including metabolic pathway, GC content and genome size by connecting to KEGG^33^ and NCBI^34^ databases. Microbial members with high abundances were involved in 134 metabolic pathways (Fig. 5A). As a functional pool, rare microbial members contributed 163 metabolic pathways and provided potential for functional compensation. Furthermore, when microbial families were classified based on the abundance patterns BV, BS or NC, microbes with BV pattern were defined to be dominant after temperature disturbance and conveyed higher GC content (Fig. 5B) and larger genome size (Fig. 5C). These observations were also conserved using the data at different taxonomic levels (Supplementary Fig. S4). It is apparent that temperature disturbance limited the growth of smaller microorganisms and encouraged microbial members to be GC-rich.

### Competitive interaction network

#### Temperature disturbance drives a competitive interaction network

**Figure 6:**
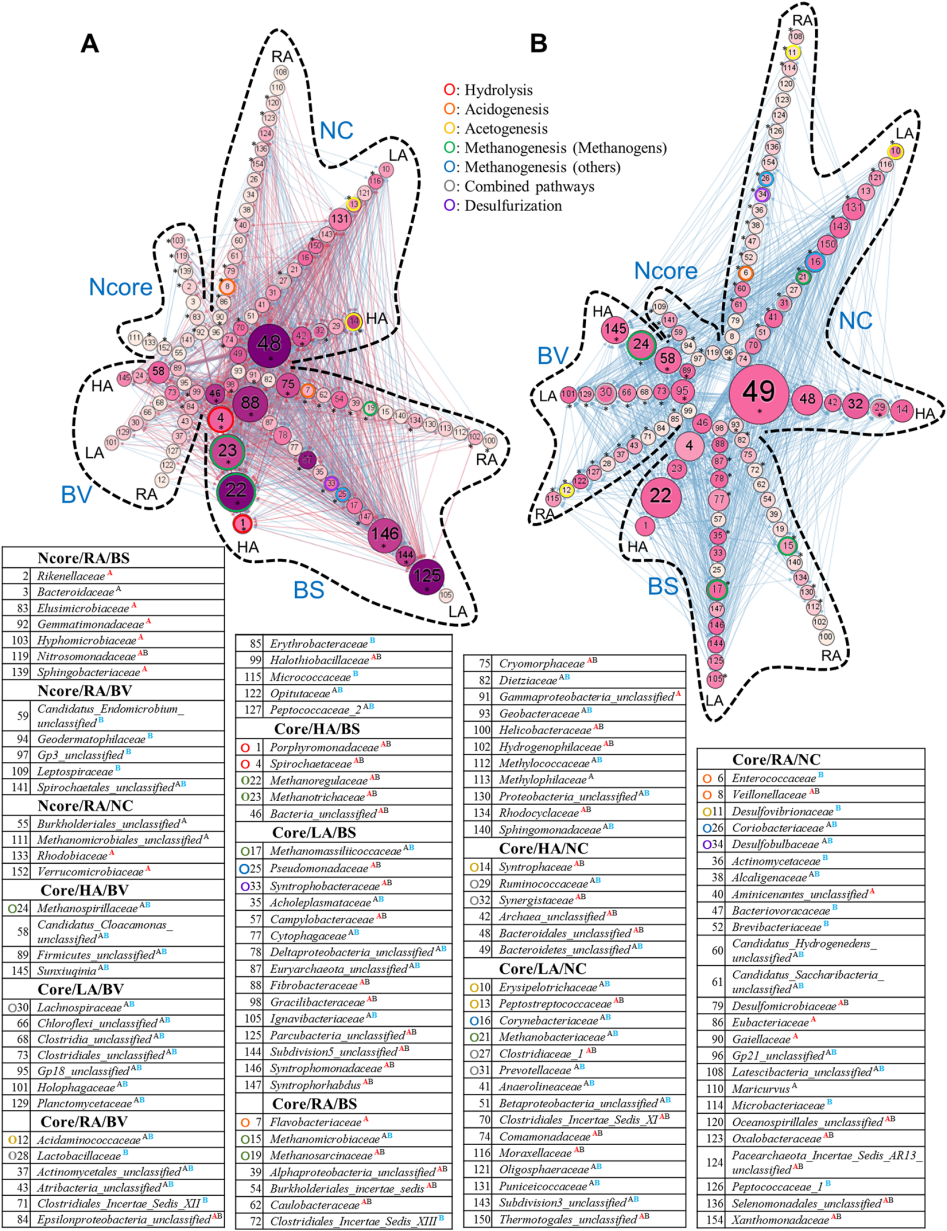
Temperature disturbance drives a competitive interaction netwotk. Microbial interaction networks (**A**) before and (**B**) after temperature disturbance are shown. Node color represents the value of in-degree centrality. The darker the node, the more the neighboring nodes influence it. Node size represents the value of betweenness centrality. The bigger the node, the more nodes that pass through it on its shortest path. Two interaction networks were compared by in-degree centrality, the larger nodes, reprsenting to obtain toplogical niche, are dennoted red A, blue B, or labelled with a star (*) in the network. For the superscript of eah microbial family, the denotation of black A (or B) ment that this microbial member could be found in the microbial network before (or after) temperature disturbance without toplogical niche.

We looked at how temperature disturbance influences microbial interactions. At the beginning, two microbial interaction networks were constructed before and after temperature disturbance and only the strongest 10% of interactions in an interaction network were retained for the network comparison (Fig. 6). There were 1,647 and 1,055 microbial interactions, containing 106 (denoted by A) and 105 (denoted by B) microbial members, respectively, predicted before and after temperature disturbance. Temperature disturbance drove a more competitive network structure with larger betweenness centrality (bigger node size) and smaller in-degree centrality (lighter node color) (Fig. 6B). Twenty-four microbial members with BS pattern seemed more influenced by other microbial members before temperature disturbance (denoted by * or red letter A in Fig. 6A). For example, *Methanoregulaceae* (Tax: 22) received 35 positive and 25 negative interactions before temperature disturbance but only 17 negative interactions after temperature disturbance. The remaining microbial families, such as *Spirochaetaceae* (Tax: 4), *Methanotrichaceae* (Tax: 23), *Fibrobacteraceae* (Tax: 88), etc., conveyed a similar pattern to *Methanoregulaceae*. Furthermore, we observed that most microbial members with the BV pattern tended to have more microbial neighbors with larger in-degree centrality after temperature disturbance other than *Epsilonproteobacteria_unclassified* (Tax: 84) and *Halothiobacillaceae* (Tax: 99). However, in the NC group, the number of microbial members with stars (*) did not dominate in any one interaction network, but they did convey a consistent trend: the most stars were in RA and least were in HA. Furthermore, temperature disturbance seemed to trigger the transformation from a subset of rare microbial members (Tax: 103, 119, 139, 2, 83, 92, 133, and 152) into a very different group (Tax: 109, 141, 59, 94, and 97).

#### The mapping of methane generation families in microbial networks

In this study, we constructed two very different microbial interaction networks before and after temperature disturbance (Fig. 6A,B). Next, we annotated methane generation pathways for microbial families. According to some results of previous investigations^23^, 28 microbial families could be assigned to five methane-related pathways: hydrolysis, acidogenesis, acetogenesis, methanogenesis, combined pathway, and desulfurization (denoted by colored circle in Fig. 6). Microbial families involved in hydrolysis, such as *Porphyromonadaceae* (Tax: 1) and *Spirochaetaceae* (Tax: 4), had higher abundances and topological niches, i.e. received more interactions, before temperature disturbance. Microbial members involved in all other methane-related pathways had different topology-niche groups before and after temperature disturbance (Fig. 6). Abundance profiles of microbial families involved in acidogenesis (Tax: 6), methanogenesis (others) (Tax: 16 and 26), and desulfurization (Tax: 34) showed similar variations, i.e. NC, and a differential topological niche (Fig. 6) after temperature disturbance. However, microbial members participating in acetogenesis, such as *Peptostreptococcaceae* (Tax: 13) and *Syntrophaceae* (Tax: 14), and combined pathway, including *Clostridiaceae_1* (Tax: 27) and *Synergistaceae* (Tax: 32), revealed similar abundance patterns (NC) and played an important role in the microbial network constructed before temperature disturbance. When focusing on the pathway of methanogenesis (methanogens), we observed that temperature disturbance made methanogens switch topological importance as described below. *Methanoregulaceae* (Tax: 22, hydrogenotrophic), *Methanotrichaceae* (Tax: 23, acetotrophic), and *Methanosarcinaceae* (Tax: 19, acetotrophic) had BS patterns and conveyed topological niche before temperature disturbance. However, after temperature disturbance, methanogens with topological niche showed heterogeneous abundance patterns: BS for *Methanomassiliicoccaceae* (Tax: 17, methylotrophic) and *Methanomicrobiaceae* (Tax: 15, hydrogenotrophic); NC for *Methanobacteriaceae* (Tax: 21, hydrogenotrophic and methylotrophic); and BV for *Methanospirillaceae* (Tax: 24, hydrogenotrophic). According to the network topology, hydrogenotrophic methanogens were predicted to change from *Methanoregulaceae* to *Methanomicrobiaceae* and *Methanospirillaceae* after temperature disturbance. Furthermore, we observed the transformation of acetotrophic (*Methanotrichaceae* and *Methanosarcinaceae*) to methylotrophic methanogens (*Methanomassiliicoccaceae* and *Methanobacteriaceae*) after temperature disturbance, indicating a change in nutritional utility for anaerobic digesters, which was hardly inferred only based on abundance profiles. Furthermore, microbial families with topological niche showed higher correlation between biogas production and microbial abundances (Supplementary Fig. S5) which supported microbial networks can reflect the influence of the representative abiotic factor.

## Discussion

The microbial community is a complex system in which diverse types of species are continuously restructuring their ecological properties or interactions in response to disturbance^6^. The present study shows that the COD and TS removal efficiency, ORP, and methane content improved after a long-term temperature disturbance, which suggests that the temperature disturbance may be a feasible strategy in shaping the profile of microbial community compositions for anaerobic digesters. These findings agree with the results of the previous studies that had an increase in COD removal rate^35^, or a decrease of total volatile fatty acids^15^ to complete the digestion of feed load after temperature shock, despite the fact that these studies used different operational factors or manure sources for anaerobic digesters^16,19^. Through our microbial, functional and network analyses, we found the complete digestion via hydrolysis process led to more complex materials being transformed into hydrogen, organic acids and methane; this was conducted by a series of reactions performed by dozens of interacting microbial populations. However, in one study, the biodegradation of organic matters did not seem influenced when the lowered temperature was restored to normal conditions^17^; this was attributed to the fact that the temperature fluctuation was short-term and so the microbial community could quickly return to its pre-disturbance composition. Another study indicated that a lower degree of temperature variation, 35 to 30 °C or 30 to 32 °C, led to a decrease in the biogas production^18^. Overall, the fluctuations in temperature influenced biogas productivity in AnD and the temperature should be maintained for AnD to progress well^36,37^. Importantly, this study serves to reinforce the understanding of various abiotic factors and to systematize the whole microbiome after temperature disturbance. In this study, there were three aspects to systematize the whole microbiome throughout microbial composition, functional change, and microbial interactions.

In spite of temperature shock, different operational factors like animal manure and organic shock loading were able to influence the quality of biogas production^17,19,38^, methane yield^15^ and methane content^38^ in AnD systems. In practice, disturbances to the microbial ecosystem, such as temperature change or feed overloading, are more likely to cause an imbalance in microbial communities and lead to digester failure. To examine the dynamics of microbial composition under the press disturbance, this study simulated a long-term temperature disturbance by interrupting normal operations under mesophilic temperature for eight days; the idea was that, after disturbance, some microbial members may disappear or change their abundance. Our findings that disparate microbial communities were generated after the temperature disturbance implies that the disturbance was strong enough to shift microbial community to a new steady state, which is remarkably consistent with Luo’s study^15^ but contrary to Gao’s findings^17^, in which the author mentioned that sudden temperature increases had little impact on the microbial community. The similar microbial composition before and after temperature shock may be that the temperature shock was only retained for one or two days and not a long term disturbance. Other related studies discuss the effects of organic overloading disturbance on anaerobic digesters. Researchers found that microbial populations could evolve differentially^16^ or remain the same after the disturbance^19^; however, the similarity in microbiota before and after temperature disturbance might be because the researchers performed analyses using higher levels of taxonomic assignment, i.e. phyla. To rapidly response to various environmental disturbances, the discovery of a microbial rare biosphere might play an important role as genetic and functional reservoirs in response to ecological processes^39^. For example, the abundance of *Elusimicrobiaceae* and *Thermotogaceae* was rare in the mesophilic anaerobic digesters but showed high at thermophilic anaerobic digester using industrial bioethanol waste as substrate^40^. Furthermore, another rare microorganism identified in current study, *Chlorobiaceae*, was found to participate in a bioprotective process to counteract the sudden disturbance of lysozyme digestion to keep sludge relative functionality stable^41^. These literatures support the idea that microbial rare biospheres played a central role in response to distinct environmental changes for reaching alternative stable states of microbial community. If a press disturbance permanently altered the microbial composition to a new stable state, we would observe a functional shift under a distinct ecosystem process. Since we found that microbial composition was changed after temperature disturbance, our next question was whether composition shifts cause functional redundancy or specificity.

The traditional way to unravel the implications of community stability and functional regime shifts in microbial ecosystem in response to press disturbance relies on specific physiological response and disturbance intensity to predict ecosystem processes^6^. In this study, we used 16S-rRNA amplicon sequencing to reveal microbial communities and PICRUST^30^ to annotate KEGG metabolic pathways^29^ and carefully checked for functional redundancy and specificity. Based on the PCA plot, we observed a significant shift in metabolic pathways in anaerobic digesters from start-up to steady-state conditions, which implies that the microbial ecosystem achieved a new steady state after temperature disturbance. In terms of genomics, we found that the microbial families after a long-term temperature disturbance had the genomic characteristics of larger genome size and higher GC content, and had a less intensive and more competitive interaction network. Microorganisms with small genomes were known to endure greater disturbances but less tolerant to competition owing to their higher growth rates and larger carrying capacities^14^. They have been found to be highly dependent on their hosts, and gene loss often disrupts functional categories of DNA repair, recombination, cell envelope biogenesis, or degradation of complex substrates^42^. Losing genes involved in DNA repair may be biased towards a new equilibrium with higher AT content, as DNA damage such as cytosine deamination or guanine oxidation occurs. Furthermore, to frequently interact with their hosts, microorganisms with reduced genomes usually maintain a subset of genes devoted to invading and persisting in host tissues, or to generate nutrients—such as essential amino acids or B vitamins—for host fitness^43^; these interactions are mutualistic relations.

This reflects that microorganisms with larger genomes are often correlated with higher GC content^9^ and have a full set of genes that fulfill all functional requirements for free living. These larger microbes were able to degrade complex substrates by themselves and easily competed with each other for nutritional requirements. Generally, microorganisms tend to shift toward genomic deletion in evolution^44^; thus, microbes must acquire additional genes, including plasmid DNA or horizontally transferred DNA, to adapt or respond to different environments^45^. Plasmids contain a few genes that show a big difference to their host’s circular chromosome in the nucleoid^46^. These genes are usually not essential for daily survival, but are able to help microbes to conquer unwonted stressful conditions. In addition, most bacteria only acquire plasmids with GC content lower than that of their own chromosome^42^. It is reasonable that microbes with large genomes usually have higher GC content and are more likely to integrate extracellular plasmids into the bacterium. In summary, we speculate that the high adaptability of the microbial community in response to extracellular environments was due to the two characteristics of larger microbial genomes: a full set of genes for microbial competition or fitness and more plasmids for microbial stress response. In previous studies, Coyte *et al*. indicates that microbial competition was able to increase ecological stability^13^ and Michael *et al*. mentions that high-degree network structure will reduce a system’s complexity^47^, which support that lower temperature is a key to making microbial communities tighter, more compact, less intensive and more competitive, thereby refining and stabilizing the microbial ecosystem. Through systematic investigations, these findings lead to the conclusion that competition relationship may play a major role in reestablishing microbial community structure throughout the disturbance.

In this study, we used the network inference method to systematize the topological changes that microbial communities made in response to temperature disturbance. Before (or after) temperature disturbance, most microbes with the BS (or BV) pattern had topological niches. It seems reasonable that microbes with the BS (or BV) pattern tend to have higher abundance and play a critical topological role in the microbial network before (or after) temperature disturbance. Incorporated with well-known pathway information, network comparison was able to detect the transition of acidogenesis-related members from *Veillonellaceae* and *Flavobacteriaceae* to *Enterococcaceae*, the transition of hydrogentrophic methanogens from *Methanoregulaceae* to *Methanomicrobiaceae*, the nutritional transition from acetotrophic (*Methanotrichaceae* and *Methanosarcinaceae*) to methylotrophic methanogens (*Methanomassiliicoccaceae* and *Methanobacteriaceae*), etc., which could not have been achieved by directly analyzing microbial compositions. This study has taken a step toward understanding the relationship between ecosystem change and a long-term disturbance, which suggests that the AnD system is a good platform for constructing short- or long-term disturbances to microbial ecosystems. The comprehensive strategies for understanding temporal ecological communities could also apply to a variety of research fields about dynamic transitions in microbial populations. Yet, some of the conclusions were drawn based on theoretical analysis, more biological experiments are required to support.

## Supplementary information

Supplementary Information

## Data Availability

NGS sequence data analyzed during this study have been deposited in the NCBI’s Short Read Archive sequence database with BioProject accessin number: PRJNA484128 (SRR7640822- SRR7640851). All data supporting the conclusions of this article are included in this published article and its supplementary information files.

## References

[1] Ebersberger D, Wermbter N, Niklaus PA, Kandeler E (2004). Effects of long term CO 2 enrichment on microbial community structure in calcareous grassland. Plant and Soil.

[2] Allison SD, Hanson CA, Treseder KK (2007). Nitrogen fertilization reduces diversity and alters community structure of active fungi in boreal ecosystems. Soil Biology and Biochemistry.

[3] Eiler A, Langenheder S, Bertilsson S, Tranvik LJ (2003). Heterotrophic bacterial growth efficiency and community structure at different natural organic carbon concentrations. Applied and environmental microbiology.

[4] Tseng CH (2013). Microbial and viral metagenomes of a subtropical freshwater reservoir subject to climatic disturbances. The ISME journal.

[5] Rinnan R, Michelsen A, Bååth E, Jonasson S (2007). Fifteen years of climate change manipulations alter soil microbial communities in a subarctic heath ecosystem. Global Change Biology.

[6] Allison SD, Martiny JB (2008). Resistance, resilience, and redundancy in microbial communities. Proceedings of the National Academy of Sciences of the United States of America.

[7] Shade A (2012). Fundamentals of microbial community resistance and resilience. Frontiers in microbiology.

[8] Foerstner KU, von Mering C, Hooper SD, Bork P (2005). Environments shape the nucleotide composition of genomes. EMBO reports.

[9] Hildebrand F, Meyer A, Eyre-Walker A (2010). Evidence of selection upon genomic GC-content in bacteria. PLoS genetics.

[10] Naya H, Romero H, Zavala A, Alvarez B, Musto H (2002). Aerobiosis increases the genomic guanine plus cytosine content (GC%) in prokaryotes. Journal of molecular evolution.

[11] Rocha EP, Danchin A (2002). Base composition bias might result from competition for metabolic resources. TRENDS in Genetics.

[12] Moran NA (2002). Microbial minimalism: genome reduction in bacterial pathogens. Cell.

[13] Coyte KZ, Schluter J, Foster KR (2015). The ecology of the microbiome: Networks, competition, and stability. Science.

[14] Violle C, Pu Z, Jiang L (2010). Experimental demonstration of the importance of competition under disturbance. Proceedings of the National Academy of Sciences of the United States of America.

[15] Luo G (2015). New steady-state microbial community compositions and process performances in biogas reactors induced by temperature disturbances. Biotechnology for biofuels.

[16] Goux X (2015). Microbial community dynamics in replicate anaerobic digesters exposed sequentially to increasing organic loading rate, acidosis, and process recovery. Biotechnology for biofuels.

[17] Gao WJ, Leung KT, Qin WS, Liao BQ (2011). Effects of temperature and temperature shock on the performance and microbial community structure of a submerged anaerobic membrane bioreactor. Bioresource technology.

[18] Chae KJ, Jang A, Yim SK, Kim IS (2008). The effects of digestion temperature and temperature shock on the biogas yields from the mesophilic anaerobic digestion of swine manure. Bioresource technology.

[19] Liu A-C, Chou C-Y, Chen L-L, Kuo C-H (2015). Bacterial community dynamics in a swine wastewater anaerobic reactor revealed by 16S rDNA sequence analysis. Journal of biotechnology.

[20] Yu Y, Lee C, Kim J, Hwang S (2005). Group-specific primer and probe sets to detect methanogenic communities using quantitative real-time polymerase chain reaction. Biotechnology and bioengineering.

[21] Federation, W. E. & Association, A. P. H. Standard methods for the examination of water and wastewater. *American Public Health Association (APHA): Washington*, *DC*, *USA* (2005).

[22] Sundberg C (2013). 454 pyrosequencing analyses of bacterial and archaeal richness in 21 full-scale biogas digesters. FEMS microbiology ecology.

[23] Shaw GT, Liu AC, Weng CY, Chou CY, Wang D (2017). Inferring microbial interactions in thermophilic and mesophilic anaerobic digestion of hog waste. PloS one.

[24] Magoc T, Salzberg SL (2011). FLASH: fast length adjustment of short reads to improve genome assemblies. Bioinformatics.

[25] Schloss PD (2009). Introducing mothur: open-source, platform-independent, community-supported software for describing and comparing microbial communities. Applied and environmental microbiology.

[26] Kozich JJ, Westcott SL, Baxter NT, Highlander SK, Schloss PD (2013). Development of a dual-index sequencing strategy and curation pipeline for analyzing amplicon sequence data on the MiSeq Illumina sequencing platform. Applied and environmental microbiology.

[27] Edgar RC (2013). UPARSE: highly accurate OTU sequences from microbial amplicon reads. Nature methods.

[28] Angly, F. E. *et al*. CopyRighter: a rapid tool for improving the accuracy of microbial community profiles through lineage-specific gene copy number correction. *Microbiome* **2**, 10.1186/2049-2618-2-11 (2014). 10.1186/2049-2618-2-11 PMC4021573 24708850

[29] Ogata H (1999). KEGG: Kyoto Encyclopedia of Genes and Genomes. Nucleic acids research.

[30] Langille MG (2013). Predictive functional profiling of microbial communities using 16S rRNA marker gene sequences. Nature biotechnology.

[31] Shaw GT, Pao YY, Wang D (2016). MetaMIS: a metagenomic microbial interaction simulator based on microbial community profiles. BMC bioinformatics.

[32] Bastian M, Heymann S, Jacomy M (2009). Gephi: an open source software for exploring and manipulating networks. ICWSM.

[33] Kanehisa M, Furumichi M, Tanabe M, Sato Y, Morishima K (2017). KEGG: new perspectives on genomes, pathways, diseases and drugs. Nucleic acids research.

[34] Coordinators NR (2017). Database resources of the national center for biotechnology information. Nucleic acids research.

[35] Ahn J, Forster C (2002). The effect of temperature variations on the performance of mesophilic and thermophilic anaerobic filters treating a simulated papermill wastewater. Process Biochemistry.

[36] Bouallagui H (2004). Effect of temperature on the performance of an anaerobic tubular reactor treating fruit and vegetable waste. Process Biochemistry.

[37] Alvarez, J., Zapico, C., Gomez, M., Ruiz, I. & Soto, M. Anaerobic treatment and pre-treatment of municipal wastewater at low ambient temperature. In *Proceedings of the 9th World Congress Anaerobic Digestion*. *Belgium*: *IWA Antwerpen*. 2–6 (2001).

[38] Peck MW, Skilton JM, Hawkes FR, Hawkes DL (1986). Effects of temperature shock treatments on the stability of anaerobic digesters operated on separated cattle slurry. Water Research.

[39] Lynch MD, Neufeld JD (2015). Ecology and exploration of the rare biosphere. Nature reviews. Microbiology.

[40] Roske I (2014). Microbial community composition and dynamics in high-temperature biogas reactors using industrial bioethanol waste as substrate. Appl Microbiol Biotechnol.

[41] Xin XD, He JG, Qiu W, Tang J, Liu TT (2015). Microbial community related to lysozyme digestion process for boosting waste activated sludge biodegradability. Bioresource technology.

[42] Nishida H (2012). Evolution of genome base composition and genome size in bacteria. Frontiers in microbiology.

[43] McCutcheon JP, Moran NA (2011). Extreme genome reduction in symbiotic bacteria. Nature reviews. Microbiology.

[44] Mira A, Ochman H, Moran NA (2001). Deletional bias and the evolution of bacterial genomes. Trends in genetics: TIG.

[45] Nishida H (2012). Comparative analyses of base compositions, DNA sizes, and dinucleotide frequency profiles in archaeal and bacterial chromosomes and plasmids. International journal of evolutionary biology.

[46] Badrinarayanan A, Le TB, Laub MT (2015). Bacterial chromosome organization and segregation. Annual review of cell and developmental biology.

[47] Hadley MW, McGranaghan MF, Willey A, Liew CW, Reynolds ER (2012). A new measure based on degree distribution that links information theory and network graph analysis. Neural systems & circuits.

