# Supplementary material for: A systematic approach re-analyzing the effects of temperature disturbance on the microbial community of mesophilic anaerobic digestion
Source: Sci Rep. 2019 Apr 25;9:6560. doi: 10.1038/s41598-019-42987-0 (PMC6484075; doi:10.1038/s41598-019-42987-0)
Supplement: Supplementary file 1 — Supplementary Information [file 41598_2019_42987_MOESM1_ESM.pdf]

A systematic approach re-analyzing the effects of temperature disturbance on the microbial community of mesophilic anaerobic digestion

Grace Tzun-Wen Shaw[1], Chieh-Yin Weng[1], Cheng-Yu Chen[1], Francis Cheng-Hsuan Weng[1] and Daryi Wang[1]*

[1]Biodiversity Research Center, Academia Sinica, Taipei 115, Taiwan

* Corresponding author
Email: DW: dywang@gate.sinica.edu.tw

Supplementary Information

Table S1 The abiotic and biotic factors before and after temperature disturbance. All measurements are represented by mean values with the standard error of the mean. F test for the equality of two variants was conducted. Two sample Student's one-tailed t tests with equal (denoted as E) and uneqaul (denoted as UE) variance were used to calculate the significance before and after temperature disturbance.

	**Before disturbance (day 1 to 24)**	**After disturbance (day 33 to 67)**	**P value (F test)**	**P value (Student's one-tailed t test)**
**ORP (mV)**	-496.47±1.67	-508.83±0.53	5.96×10^{-7}	UE: 6.18×10^{-8}
**CH_4 %**	45.73±2.48	55.06±0.78	6.42×10^{-7}	UE: 6.34×10^{-4}
**GPR (L/Lday)**	0.94±0.10	0.81±0.03	9.28×10^{-9}	UE: 0.118
**MPR (L/Lday)**	0.40±0.04	0.44±0.01	1.80×10^{-5}	UE: 0.179
**RCOD (%)**	7.76±2.01	29.31±1.36	0.258	E: 1.62×10^{-12}
**RTS (%)**	10.36±1.64	13.54±0.79	0.006	UE: 0.046
**Shannon index**	2.77±0.10	2.84±0.07	0.33	E: 0.28
**N(microbial families)**	114.83±1.45	103.47±1.01	0.37	E: 3.09×10^{-7}
**Archaea %**	15.00±1.70	13.75±0.41	2.67×10^{-5}	UE: 0.24

Table S2 The partitioned 154 microbial families. There are three mannors to separate microbial members, such as the level of abundance (HA/LA/RA), abundance patterns (BS/BV/NC) and the degree present (Core/Ncore).

		BS	BV	NC
Core	HA	5	4	6
	LA	15	7	15
	RA	18	12	26
Ncore	RA	18	6	22

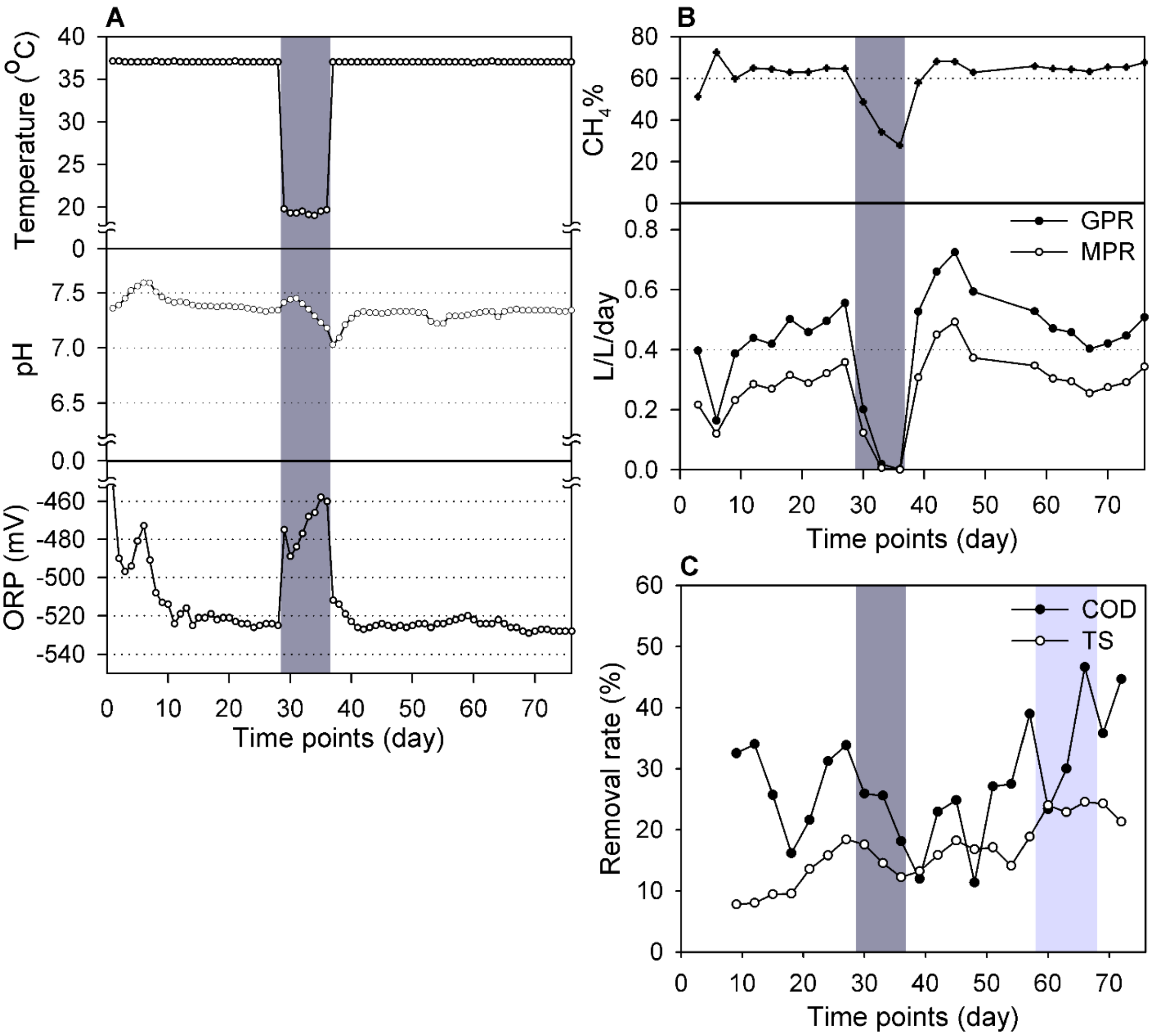

Figure S1 The replicate experiment for the temperature disturbance at the anaerobic digester revealed similar abiotic patterns. The change in (**A**) temperature, pH, ORP, (**B**) CH_4%, GPR, MPR, and (**C**) removal rate of COD, TS are consistent with the descriptions in Guo et al[15]. We reasoned that the 16rRNA data extracted from day 11 to day 20 (1st steady state), and day 52 to day 60 (2nd steady state) (Fig. 1A) have reached a steady state and are feasible for the following analysis.

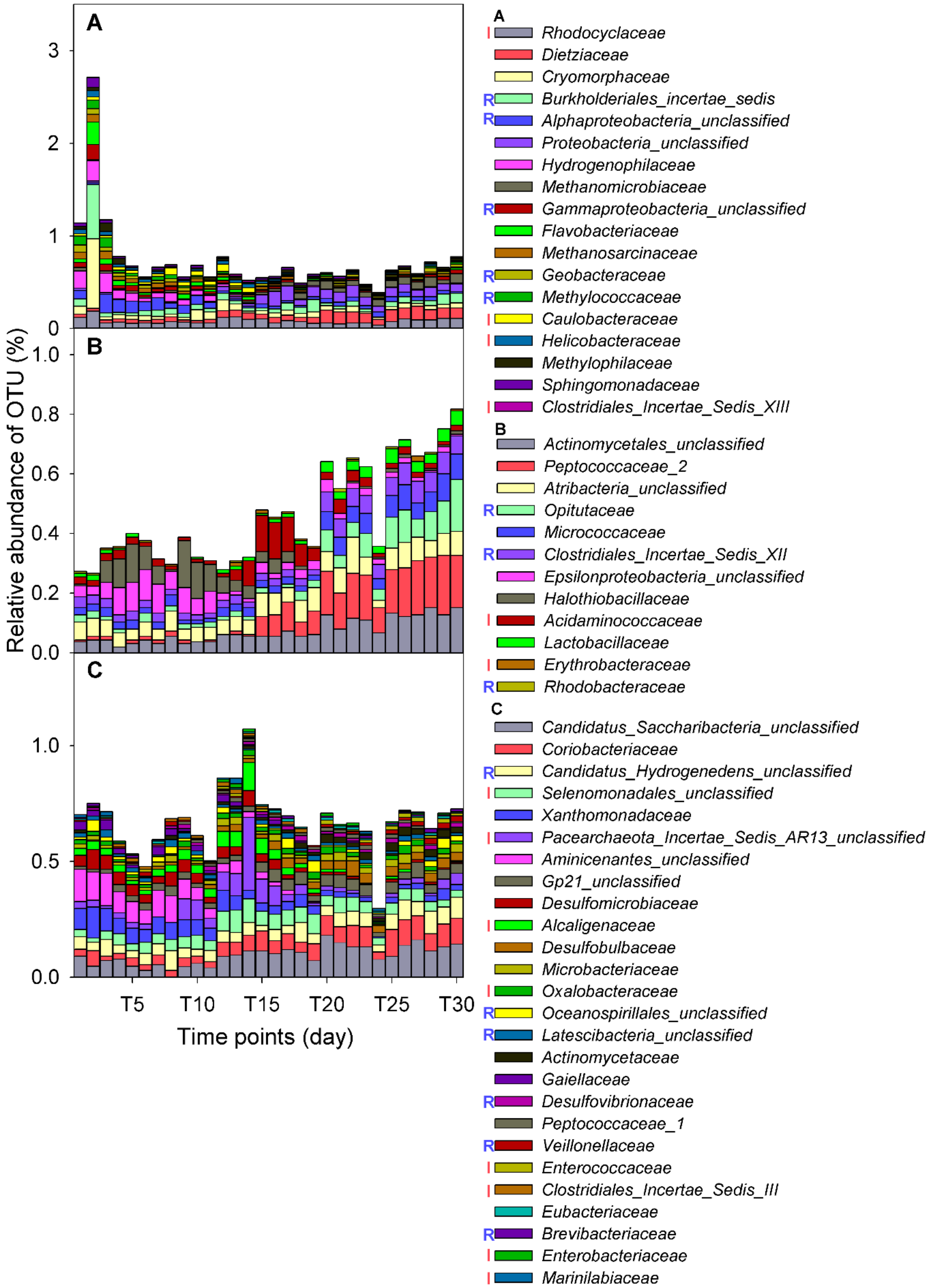

Figure S2 Microbial composition was dynamically changed and partitioned based on following criteria: (**A**) Core/RA/BS, (**B)** Core/RA/BV, and (**C**) Core/RA/NC.

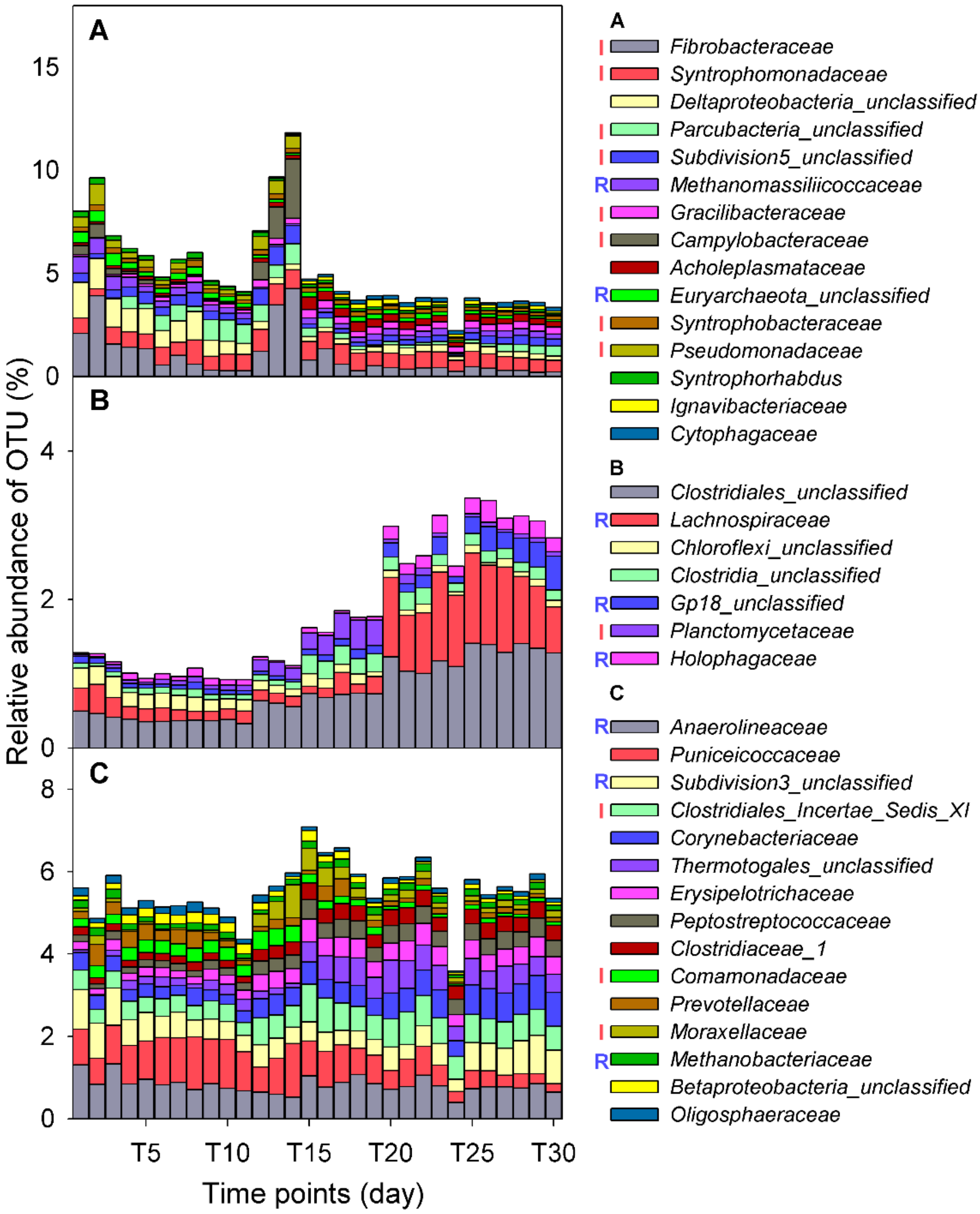

Figure S3 Microbial composition was dynamically changed and partitioned based on following criteria: (**A**) Core/LA/BS, (**B**) Core/LA/BV, and (**C**) Core/LA/NC.

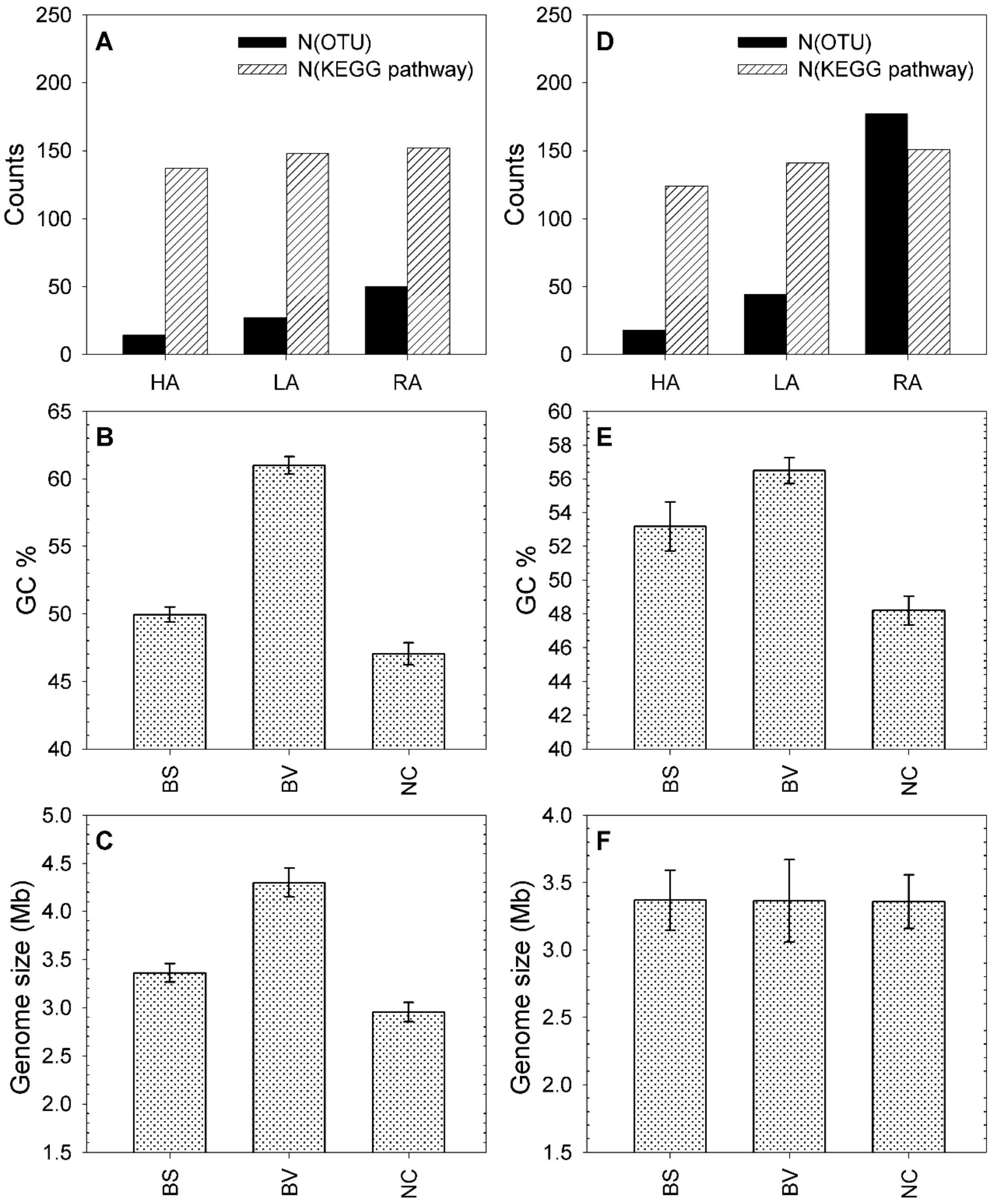

Figure S4 Metabolic pathaway and genomic properties at order (**A**, **B** and **C**) and genus (**D**, **E** and **F**) level for number of microbial families, number of KEGG pathways, GC content (GC%) and genome size of each abundance pattern. The results were consistent with the observations in Fig 5, excepting (F), it is likely that the numbers calculated in genus level are very diverse.

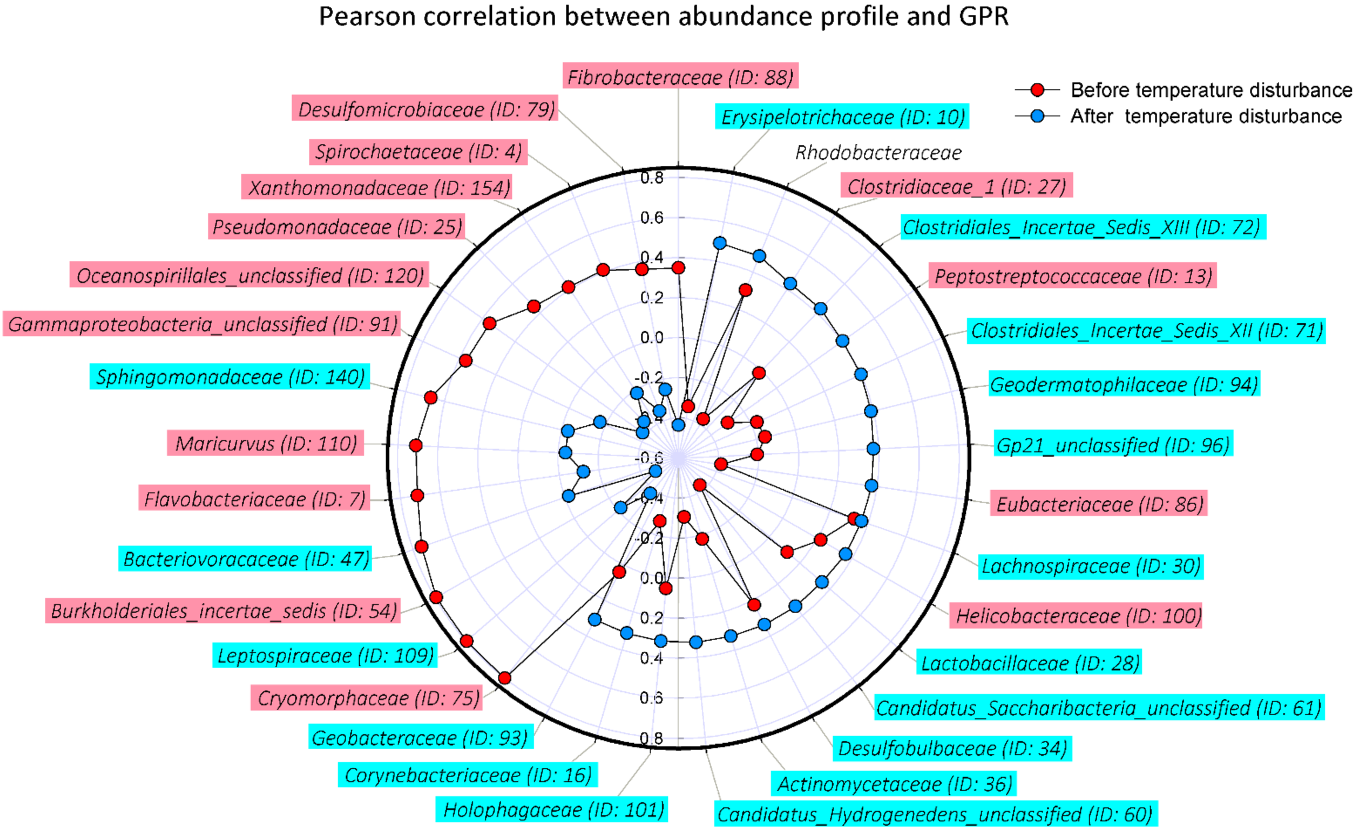

Figure S5 Microbial members with topoligical nich had positive correlation with higher GPR before and afer disturbance. For each microbial family, two pearson correlations between abundance profiles and GPR were calculated. The fist correlation between GPR and 12 time-series abundances before temperature disturbance were denoted by red circles (●). There were 15 time points for the second correlation which was denoted by blue ones (●).Taken the first correlation as reference, we picked up 14 microbial members with higher pearson correlation (≥ 0.3). If the second correlation (≥ 0.3) was used to select microbes, there were 19 microbial families shown to have higher correlation after temperature disturbance. A microbial member marked as pink red (or cyan blue) had topological niche before (or after) temperature disturbance in Fig. 6A (or Fig. 6B).
